# The Modern Treatment of Syphilitic Diseases, Both Primary and Secondary

**Published:** 1840-01

**Authors:** 


ART. III.—*The Modern Treatment of Syphilitic Diseases, both primary and secondary: comprising an Account of the New Remedies, with Numerous Formulæ for their Preparation and Mode of Administration.* By LANGSTON PARKER, M.R.C.S.—*London*, 1839. 12mo, pp. 158.

FROM the title of Mr. Parker's work, "On the Modern Treatment of Syphilitic Diseases," we had expected a summary of the opinions of British as well as Foreign surgeons on this important point; but the author has almost wholly confined himself to the treatment adopted at present by the French. This task Mr. Parker has accomplished most creditably. His book is carefully compiled, concise, clearly expressed, and well adapted to put the English reader in possession (in a short compass) of the opinions and practice of the principal surgeons attached to the venereal hospitals of Paris. Cullerier, Desruelles, and Ricord are the chief authorities quoted, also the late Dr. Wallace, of Dublin; at the same time, Mr. Parker has "added the result of his own experience to the weight of others." We had marked for extract Mr. Parker's excellent account of M. Ricord's experiments on the inoculation of pus from venereal sores, but we are constrained, by want of room, to omit them. They add considerably to our precise knowledge of the natural history of the disease, and merit the attention of all surgeons.

Mr. Parker himself is inoculated with the French love of leeches. They are to be applied to the centre of venereal ulcers, to their thickened edges, to the glans on each side of the prepuce in gonorrhœa, indeed to all manner of places which have usually been avoided, and on this account seem to have been selected. "The only objection," says Mr. P., with much naïveté, "to this practice, is the inoculation of the leech-bites, and, consequently, the formation of fresh phagedænic sores." (p. 97.)

For details of modern practice in general, this work is a useful guide; it contains numerous formularies for the new combinations of mercury and iodine, and other recently-introduced medicines. Some valuable practical hints may be gathered from it, on the expediency of the mercurial treatment.

In the French venereal hospitals, mercury is not used in the treatment of primary venereal sores (p. 12); but yet Ricord, Cullerier, and Desruelles, surgeons of those hospitals, admit the superior efficacy of mercury in hastening the cicatrization of a primary venereal ulcer, and in diminishing the risk of secondary symptoms. They are all three partisans of the simple treatment (the non-mercurial); but they recommend mercury "when the sore is indolent, does not cicatrize under the simple plan; when its edges are hard and elevated, or the sore leaves behind it, in healing, an indurated cicatrix." We believe that this is much the practice of judicious surgeons in this country; it is certainly that which is recommended by the highest surgical authorities. The simple plan, we believe, has been confined almost to hospitals, where the patients can be compelled to rest and low diet; but in private practice, where this is often impossible, all surgeons have found the necessity of some more active measures; and mercury, although it may be denied by some to be a specific against syphilis, yet is allowed by all to be one of the most powerful therapeutic agents we can employ.

If a mere routine practice is pursued, without any reference to the kind of sore, it is not improbable, that less harm will be done by the non-mercurial treatment than the mercurial. Riddled and honeycomb skulls, looking as if they had been eaten of worms and gnawed by dogs, are now, fortunately, rarely to be seen, except in the glass-cases and drawers of surgical museums, or in Cheselden's plates;—venerable relics of a past age, out of date, like the silk coat and full ruffles of the contemporaneous doctor. These were the consequences of a profuse abuse of mercury, which is now rarely attempted. But still, simple excoriations, herpes præputialis, and superficial sores, the joint product of abrasion and want of cleanliness, are often treated by mercury; the healthy constitution being thus cruelly subjected to the long-continued action of a powerful poison. Such instances have often fallen under our own observation, and are doubtless common. There is another abuse, which is perhaps more common, from mercury being given irregularly, or in insufficient quantity, without any attention either to the habit of body, which may be either weak or scrofulous, and without precautions in preserving the patient from wet and cold. An obstinate disease is thus generated, which John Hunter, with great clearness and brevity, has defined as the joint effect of the poison, the constitution, and the remedy. By the routine adoption of the non-mercurial plan, these cases would not occur; and if we may believe the experiments, although secondary symptoms would follow more frequently, they would be less severe and intractable. But this supposes the abuse of mercury, not whether its judicious employment, due regard being had to the kind of sore, the constitution, and to precautionary measures, is not, as a general rule, the most expedient in practice. We believe that it is, and that in private practice especially, no other can be adopted.

